# Blood-Based Protein Biomarker Panel for the Detection of Colorectal Cancer

**DOI:** 10.1371/journal.pone.0120425

**Published:** 2015-03-20

**Authors:** Kim Y. C. Fung, Bruce Tabor, Michael J. Buckley, Ilka K. Priebe, Leanne Purins, Celine Pompeia, Gemma V. Brierley, Trevor Lockett, Peter Gibbs, Jeanne Tie, Paul McMurrick, James Moore, Andrew Ruszkiewicz, Edouard Nice, Timothy E. Adams, Antony Burgess, Leah J. Cosgrove

**Affiliations:** 1 CSIRO Preventative Health National Research Flagship, Adelaide, South Australia, Australia; 2 Royal Melbourne Hospital, Melbourne, Victoria, Australia; 3 Cabrini Hospital, Melbourne, Victoria, Australia; 4 Royal Adelaide Hospital, Adelaide, South Australia, Australia; 5 SA Pathology, Adelaide, South Australia, Australia; 6 Monash University, Melbourne, Victoria, Australia; 7 CSIRO Materials Science & Engineering, Parkville, Victoria, Australia; 8 Walter and Eliza Hall Institute for Medical Research, Parkville, Victoria, Australia; 9 Department of Surgery, University of Melbourne, Royal Melbourne Hospital, Victoria, Australia; Deutsches Krebsforschungszentrum, GERMANY

## Abstract

**Background:**

The majority of colorectal cancer (CRC) cases are preventable by early detection and removal of precancerous polyps. Even though CRC is the second most common internal cancer in Australia, only 30 per cent of the population considered to have risk factors participate in stool-based test screening programs. Evidence indicates a robust, blood-based, diagnostic assay would increase screening compliance. A number of potential diagnostic blood-based protein biomarkers for CRC have been reported, but all lack sensitivity or specificity for use as a stand-alone diagnostic. The aim of this study was to identify and validate a panel of protein-based biomarkers in independent cohorts that could be translated to a reliable, non-invasive blood-based screening test.

**Principal Findings:**

In two independent cohorts (n = 145 and n = 197), we evaluated seven single biomarkers in serum of CRC patients and age/gender matched controls that showed a significant difference between controls and CRC, but individually lack the sensitivity for diagnostic application. Using logistic regression strategies, we identified a panel of three biomarkers that discriminated between controls and CRC with 73% sensitivity at 95% specificity, when applied to either of the two cohorts. This panel comprised of Insulin like growth factor binding protein 2 (IGFBP2), Dickkopf-3 (DKK3), and Pyruvate kinase M2(PKM2).

**Conclusions:**

Due to the heterogeneous nature of CRC, a single biomarker is unlikely to have sufficient sensitivity or specificity for use as a stand-alone diagnostic screening test and a panel of markers may be more effective. We have identified a 3 biomarker panel that has higher sensitivity and specificity for early stage (Stage I and -II) disease than the faecal occult blood test, raising the possibility for its use as a non-invasive blood diagnostic or screening test.

## Introduction

Colorectal cancer (CRC) is the third most common cancer type diagnosed worldwide where it constitutes approximately 10% of all cancer diagnoses and has an estimated annual mortality rate over 600,000[1]. Countries such as Australia, New Zealand, Europe, the US, and the UK are reported to have the highest incidence of disease, however, the incidence is increasing in countries such as Japan and those of Eastern Europe. The majority of cases (up to 80%) are sporadic where environmental and lifestyle factors are believed to play a role in its development [2]. CRC is a heterogeneous disease which develops via an accumulation of genetic mutations and epigenetic changes in the colonic epithelium that eventually results in neoplastic transformation [3–5]. The slow and progressive nature of this process presents an opportunity to implement screening programs and diagnostic tools for the early detection of the disease that have the potential to reduce incidence and mortality associated with CRC. In an attempt to reduce incidence and to detect the disease in its early stages before symptoms are evident, screening programs have been implemented in many countries including the US, UK, Australia, Japan, and Europe [6–8].

Currently, the most widely used diagnostic tools include endoscopic procedures such as colonoscopy and sigmoidoscopy, and the guaiac-based faecal occult blood test (gFOBT) or the immunochemical faecal occult blood test (iFOBT), also known as the faecal immune test (FIT)[9]. While colonoscopy and sigmoidoscopy are the most sensitive procedures for detection of colorectal tumours and precancerous lesions (adenomas and polyps), and can be potentially curative if polyps are removed, these procedures are difficult to implement on a population-wide basis due to cost, invasiveness, expertise required, and time-consuming nature due to the bowel preparation required [10,11]. While cheap and non-invasive, the FOBT and FIT have lower sensitivity and specificity than colonoscopy (i.e., these tests have higher false positive rates) and are most successful at detecting late stage disease [10,12]. Furthermore, the diagnostic performance of FOBT and FIT is variable, with reported sensitivities for CRC between 11–64% for gFOBT (79–98% specificity) and 56–89% for FIT (83–97% specificity)[13]. These tests are also often compromised by poor patient compliance, variations in analytical procedures such as different methods of stool collection and handling, the need for multiple test samples, and variations in the interpretation of test results [13,14]. Currently, only the gFOBT has been shown to reduce CRC mortality in large prospective randomised clinical trials [8,13].

Many studies have been published reporting biomarkers that can be implemented as a non-invasive test to detect CRC, especially in its early stages (Stage I and/or premalignant disease). A number of strategies for identifying blood-based protein biomarkers have been reported in the literature, including proteomic and/or gene expression analysis of colorectal tumour tissue and secreted proteins. Although some of these studies have identified potential panels of proteins or genes suitable for CRC detection [15–19], data from follow-up studies, for instance in larger patient cohorts, are not available. Currently, the most promising biomarkers appear to be DNA methylation markers, including methylated septin 9 (mSEPT9) measured in blood [20–23] and a stool-based DNA test consisting of methylated BMP3, NDRG4, VIM, and TFPI2 and a mutant form of KRAS[24]. More recently, a modified stool-based DNA test consisting of methylated BMP3 and NDRG4, mutant KRAS and ß-actin as the control in combination with faecal haemoglobin was tested in an asymptomatic screening population consisting of 9,989 patients [25]. It was noted that the DNA test had lower specificity, a higher false positive rate and suffered from a higher technical failure rate due to its complex nature which may hamper its implementation as a population wide screening test. Although stool-based DNA tests have shown promising results for CRC detection in clinical trials, they are yet to be implemented into clinical practice or as a population-wide screening test.

Due to the heterogeneous nature of CRC, a single biomarker is unlikely to have sufficient sensitivity or specificity for use as a stand-alone diagnostic screening test and a panel of markers may be more effective. Previously, we evaluated the performance and suitability of 32 protein biomarkers in the serum and/or plasma of colorectal cancer patients and normal controls [26]for their ability to diagnose CRC. Although this analysis identified 12 protein biomarkers that differed significantly between the two groups, no one protein had adequate sensitivity and specificity for use as a stand-alone diagnostic. We also identified potential biomarker combinations representing different aspects of the disease process that could lead to a diagnostic test for CRC. Here we report on the evaluation of seven of these protein biomarkers (IGFBP2, PKM2, DKK3, MAC2BP, tissue inhibitors of metalloproteinases 1 (TIMP1), Interleukin 8 (IL8) and Interleukin 6 (IL6) as a potential diagnostic or screening test for CRC.

## Materials and Methods

### Ethics Statement

All research protocols used in this study was approved by the relevant Human Research Ethics Committees at Commonwealth Scientific Industrial Research Organisation, Adelaide (CFNS Human Research Ethics Committee-Proposal 03/17 (a) & (b)), and the Royal Melbourne Hospital, Melbourne (HREC project 2003.145 & 2003.146). Written informed consent was obtained from each patient prior to blood sample collection.

### Sample collection

Patients were newly diagnosed cases of CRC with no previous history of disease. Blood was obtained prior to surgery at colorectal surgery preadmission clinics from a network of hospitals associated with the Victorian Cancer Biobank, Melbourne, Victoria, Australia, between 2005 and 2011. Patients who had already received chemo- and/or radio- therapy were excluded from this study. For CRC patients, blood samples were taken after diagnosis and at least one day before surgery. Staging was conducted according to the TNM classification for colon and rectal cancer [27].

Serum samples from CRC patients and age/gender matched controls were collected using a standard operating procedure as previously described [28,29]. Blood was collected into serum gel tubes (Scientific Specialties Inc., USA) and each sample was left to stand at room temperature for 30 min prior to centrifugation (1,200g, 10 min, room temperature). The serum fraction was then transferred to clean 15 mL tubes and centrifuged again (1,800g, 10 min, room temperature) prior to being aliquotted (250 μL) and stored (−80°C). All samples were processed and stored within 2 hrs of collection.

### Biomarker analysis and identification of the biomarker panel

The following biomarkers were measured using commercially available ELISA kits according to the respective manufacturer protocols: IGFBP2 (Diagnostic Systems Laboratories, USA or Demeditec Diagnostics GmbH, Germany), MAC2BP (Bender MedSystems GmbH, Austria), PKM2 (Schebo Biotech, Germany), DKK3 (R&D Systems, USA) and TIMP1 (R&D Systems, USA). IL6 and IL8 were analysed as bead-based assays sourced from R&D Systems (Minneapolis, MN, USA). For each assay, samples were measured in duplicate and in-house quality control (QC) samples were included. QC samples consisted of a pooled normal sample (n = 41) and pooled CRC patient sample (n = 41). For each assay, the inter- and intra-assay coefficients of variation (CV) were less than 10%. This is consistent with the manufacturer specifications.

For the standard ELISAs, the absorbance or fluorescence signal was detected using the Wallac Victor^3^ 1420 multilabel counter (Perkin Elmer, USA). Biomarker concentrations were derived from the respective standard curve using the WorkOut software (version 2.0). For IL8 and IL6, preliminary data was analysed using the Luminex IS2.3 software (Qiagen, Hilden, Germany).

The Prism software package (v6, Graphpad Software Inc., San Diego, CA, USA) and the R statistical software packages were used for statistical analysis. The non-parametric Wilcoxon rank sum test was used to determine the statistical difference between cancer and control patients, and receiver operator characteristic (ROC) curve analysis was performed to assess the diagnostic performance for each marker and to determine the sensitivity for each at 95% specificity. Bootstrap confidence intervals with 20,000 bootstrap resamples for area under the curve (AUC) was performed using the R package pROC [30].

Biomarkers were selected for the panel using forward stepwise variable selection and Bayesian information criterion (BIC) penalty to prevent over-fitting. This process of variable selection and estimation of coefficients was performed in Cohort 1 (training data set) and then to Cohort 2 (test data set). The model was then applied to both cohorts to identify the best performing panels that cross validated.

## Results

### Performance of individual biomarkers measured in the serum of CRC and control patients

The clinical characteristics for the patient cohorts are shown in Table 1. The levels of all seven proteins differed significantly between the patient and control groups in both the training and test cohorts (Table 2 and S1 Fig.). With the exception of DKK3, all markers were elevated in the patient group. ROC analysis was also performed to determine the ability of each protein to discriminate between the patient and control groups (Table 3 and S2 Fig.). PKM2 was the best performing biomarker with a sensitivity of 56% (p<0.0001) and 59% (p<0.0001) at 95% specificity for CRC overall when measured in the training and test cohorts, respectively. Similarly, PKM2 also proved to be the most successful marker at identifying CRC at each disease stage when compared to the control population in this particular study, including early stage disease (sensitivities of 48% (p = 0.0008), 52% (p<0.0001), 61% (p<0.0001) and 75% (p<0.0001) for Stages I, II, III and IV, respectively in the training cohort and sensitivities of 52% (p<0.0001), 65% (p<0.0001), 54% (p<0.0001) and 80% (p<0.0001) for Stages I, II, III and IV, respectively in the test cohort) (S1 Table). The performance characteristics of individual biomarkers in the training and test cohorts can be found in S1 Table. When considered individually, none of these biomarkers had sufficient sensitivity to diagnose CRC.

**Table 1:** Characteristics of the colorectal cancer and normal patients used in this study cohort.

	Cohort 1 (Training data set)		Cohort 2 (Test data set)	
Characteristics	Control	Colorectal cancer	Control	Colorectal cancer
**N**	50	95	99	98
**Gender, N**				
**Female**	25	50	33	34
**Male**	25	45	66	64
**Median age, yrs (range)**	70 (50–85)	67 (44–93)	69 (36–89)	67 (25–89)
**AJCC TNM stage**				
**I**		21		27
**II**		31		31
**III**		33		28
**IV**		10		12

**Table 2:** Concentration (median and range) for individual protein biomarkers measured in the serum of cancer and control patients.

	Cohort 1 (Training data set)			Cohort 2 (Test data set)		
	Control	Colorectal cancer	P value	Control	Colorectal cancer	P value
**PKM2 (U/mL)**	80.16 (31.20–171.2)	161.2 (32.72–392.3)	<0.0001	46.43 (15.16–125.8)	127.3 (29.78–345.8)	<0.0001
**IL6 (pg/mL)**	1.210 (0.2700–4.740)	1.745 (0.5–55.80)	<0.0001	1.590 (0.2500–48.98)	2.850 (0.3800–186.9)	<0.0001
**DKK3 (pg/mL)**	37407 (20714–529848)	30303 (10367–353232)	0.0004	32169 (13775–144377)	28354 (11208–94505)	0.0042
**IL8 (pg/mL)**	11.26 (4.360–49.89)	15.75 (3.710–103.5)	0.0006	9.735 (4.640–41.73)	16.05 (4.240–675.5)	<0.0001
**IGFBP2 (ng/mL)**	430.3 (132.9–1029)	513.1 (186.0–9347)	0.0006	469.2 (137.6–1206)	554.2 (135.0–2031)	0.0121
**Mac2BP (ng/mL)**	7126 (3918–20150)	8350 (4290–40870)	0.0008	4987 (1842–29691)	6481 (2568–20218)	<0.0001
**TIMP1 (ng/mL)**	166.6 (126.4–248.7)	187.1 (101.0–497.6)	0.0235	184.9 (107.0–315.5)	205.9 (121.1–875.2)	0.0002

**Table 3:** Classification performance of the seven protein biomarkers in the training and test cohorts.

	Cohort 1 (Training data set)				Cohort 2 (Test data set)			
	AUC	p value	Sensitivity (%) at 95% specificity	Cutoff	AUC	p value	Sensitivity (%) at 95% specificity	Cutoff
**PKM2 (U/mL)**	0.82 (0.76–0.85)	<0.0001	56	>140.7	0.91 (0.88–0.94)	<0.0001	59	>107.9
**IL6 (pg/mL)**	0.70 (0.61–0.76)	0.0002	27	>2.895	0.75 (0.67–0.80)	<0.0001	27	>4.790
**DKK3 (pg/mL)**	0.68 (0.61–0.75)	0.0004	19	<23048	0.62 (0.55–0.68)	0.0042	11	<18262
**IL8 (pg/mL)**	0.68 (0.59–0.74)	0.0006	38	>21.86	0.74 (0.71–0.79)	<0.0001	30	>24.43
**IGFBP2 (ng/mL)**	0.67 (0.59–0.76)	0.0006	21	>874.6	0.60 (0.53–0.67)	0.0123	23	>862.0
**Mac2BP (ng/mL)**	0.68 (0.59–0.75)	0.0008	35	>9304	0.70 (0.65–0.77)	<0.0001	12	>10158
**TIMP1 (ng/mL)**	0.62 (0.55–0.68)	0.0236	20	>237.2	0.65 (0.60–0.70)	0.0002	15	>265.6

**Abbreviations**: AUC, area under the receiver operating characteristic curv

### Identification of a three-biomarker panel for CRC diagnosis

**Fig 1:**
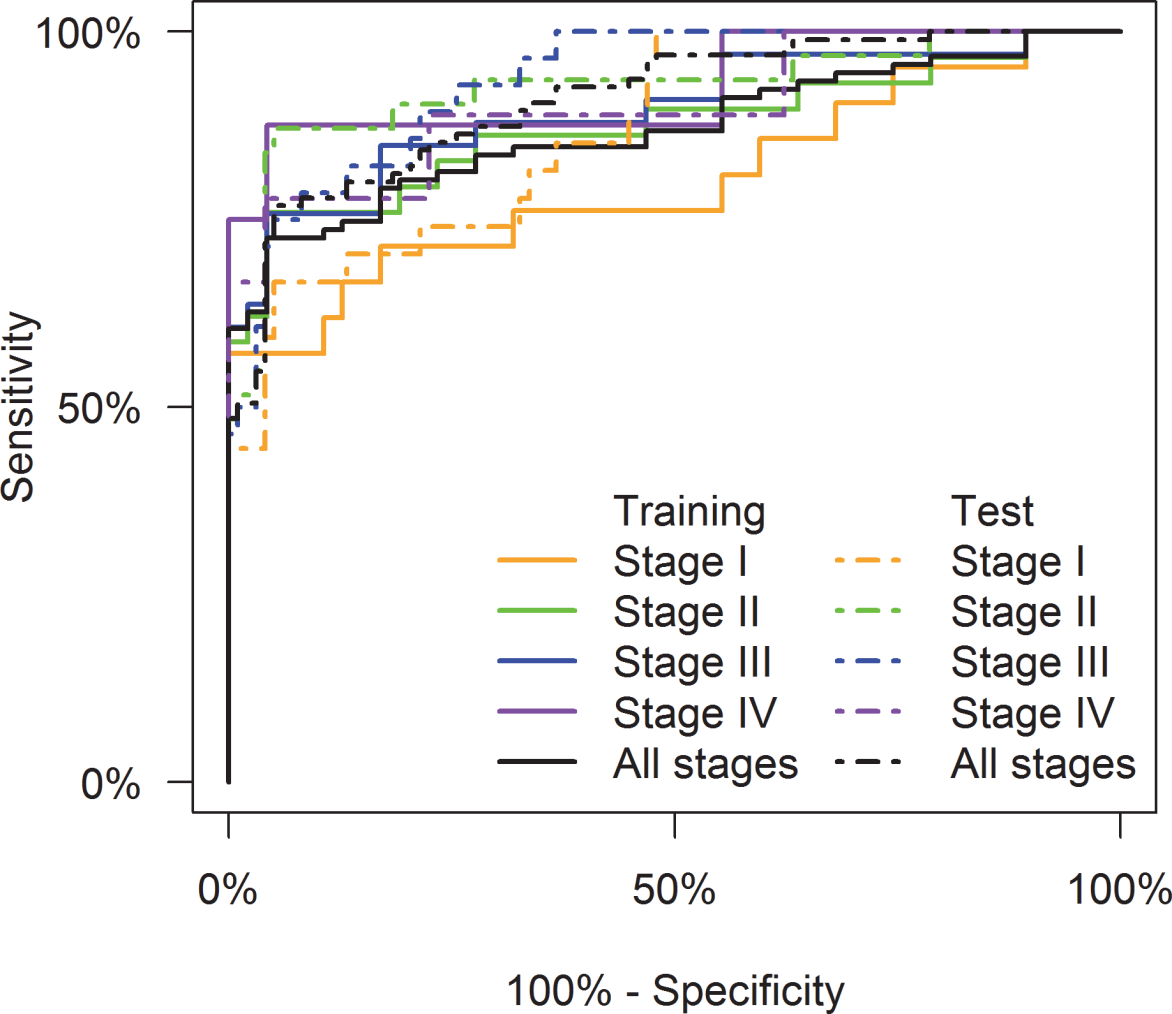
Receiver operator characteristic (ROC) curves by AJCC TNM stage for the three biomarker model, fitted to the training data, and applied to both training and test data.

Using forward stepwise logistic regression applied to the training data set (Cohort 1), a three biomarker model consisting of DKK3, PKM2 and IGFBP2 was identified that could diagnose CRC with 73% sensitivity at 95% specificity (Table 4). Furthermore, this three-biomarker model proved to be robust when validated in the test cohort (Cohort 2, sensitivity of 73% at 95% specificity), and was able to discriminate between controls and CRC patients at different TNM stage with similar sensitivity (Table 4). Importantly, this biomarker model is able to identify patients with early stage disease with high sensitivity (i.e., 57% and 76% sensitivity, at 95% specificity for Stages I and II, respectively, in the training cohort and 59% and 84% for Stages I and II, respectively, in the test cohort). Fig. 1 shows the ROC curve for the three-biomarker model and the performance characteristics of the model is detailed in Table 4.

**Table 4:** Performance characteristics of the three-biomarker model (DKK3, IGFBP2 and PKM2).

	CRC all disease stages		Stage I		Stage II		Stage III		Stage IV	
	Training	Test	Training	Test	Training	Test	Training	Test	Training	Test
**Area under the ROC curve (95% CI)**	0.87 (0.81–0.92)	0.91 (0.87–0.95)	0.80 (0.66–0.92)	0.87 (0.79–0.94)	0.87 (0.77–0.96)	0.92 (0.85–0.98)	0.90 (0.81–0.96)	0.93 (0.88–0.97)	0.93 (0.77–1.00)	0.90 (0.75–1.00)
**Sensitivity (%) at 95% specificity**	73	73	57	59	76	84	76	71	88	78
**P value**	<0.0001	<0.0001	<0.0001	<0.0001	<0.0001	<0.0001	<0.0001	<0.0001	<0.0001	<0.0001

**Abbreviations**: CRC, colorectal cancer; ROC, receiver operating characteristic

## Discussion

Previously, we measured 32 protein biomarkers in the plasma and sera of CRC patients and controls [26]. These biomarkers were initially identified as being potentially useful for CRC diagnosis based on biology, gene expression microarray and proteomic data, from both our own studies and from the literature. In our previous study, we measured PKM2 in plasma and determined that it had 19% sensitivity (at 95% specificity) whereas in serum we observed sensitivity of 56% at 95% specificity. When measured in serum, PKM2 was the best performing biomarker for CRC diagnosis when compared to the other biomarkers we measured. Furthermore, it was also the best performing marker when distinguishing early stage disease (48% sensitivity). As a further extension to this study, we have undertaken further evaluation of seven of these proteins in two independent case control cohorts (Cohort 1 and 2). Furthermore, we have identified a panel of three protein biomarkers that is able to diagnose CRC from a control population with 73% sensitivity at 95% specificity in both the training (n = 145) and test (n = 197) cohorts in our study. Although these initial studies are promising, we are currently undertaking further testing of our biomarker panel in a patient cohort which includes non-malignant colorectal diseases (e.g., inflammatory bowel disease, diverticulutis), adenomas, benign and/or precancerous polyps and other cancers. This will enable us to determine the specificity of the panel for CRC detection and its sensitivity for early stage or premalignant disease.

Our three biomarker panel consists of IGFBP2, DKK3 and PKM2, and each of these proteins are known to be biologically important in CRC disease and progression and are representative of the heterogeneous nature of this cancer. For example, there are numerous reports indicating the involvement of IGFBP2 in proliferation, migration and invasion of cancer cells [31], and elevated levels of IGFBP2 have also been reported in the serum and plasma of CRC patients when compared to normal controls [32–35]. Transcriptional silencing of DKK3 due to promoter hypermethylation in CRC tissue has been reported [36,37], and this may explain the decreased levels in the serum of CRC patients we observed in this study. However, an elevated protein expression level in the endothelial cells of microvessels of cancer tissue has also been reported indicating the potential importance of DKK3 in CRC progression, due to angiogenesis and neovascularisation [38]. PKM2 is a cytosolic enzyme involved in energy metabolism that is expressed by both normal proliferating cells and cancer cells. Elevated expression of the tumour-specific dimeric form of this protein has been reported in CRC and other gastrointestinal cancers [39–41] and numerous studies have evaluated its usefulness as a faecal or blood-based marker for CRC screening and/or diagnosis [40,42–46]. The primary disadvantage concerning its utility as a stand-alone diagnostic marker has been its poor specificity for CRC.

The performance of the three-biomarker model consisting of IGFBP2, DKK3 and PKM2 (73% sensitivity at 95% specificity) is equivalent to that quoted for FOBT and FIT (sensitivity 61–79% at 86–95% specificity) [12,14,47–49]. Furthermore, the panel of biomarkers that we have identified appears to perform well for early stage disease detection (i.e., Stage I and II disease). This superior performance at early stages, particularly at Stage I, provides an important advantage of our biomarker panel over currently used non-invasive tests for CRC. This is an important consideration since early disease detection and appropriate patient management improves overall survival for this disease. Further testing of the panel in a large prospective cohort, which includes patients with high-risk adenomas or polyps, is needed to fully understand the potential utility of our panel for diagnostic or screening purposes. It is also possible that the biomarker panel can be used in combination with current screening modalities, such as FOBT, FIT, or the recently reported plasma mSEPT9 test or stool-based DNA markers [20–24] to further improve diagnostic performance.

While it is known that screening programs can reduce mortality from CRC, emerging evidence suggests that a non-invasive blood-based test with high sensitivity and specificity for the disease, in particular early stage disease, may be advantageous to overcome perceived barriers to participation associated with the use of FOBT [50,51]. While non-invasive, the FOBT is not specific for CRC and it is not able to accurately detect early stage disease. Furthermore, the value of this test is hampered by poor patient compliance [50]. Data from studies which evaluate patient preference for blood testing versus endoscopy, including colonoscopy or sigmoidoscopy, are not available. However, studies investigating patient preference between FOBT and colonoscopy show conflicting results. For instance, Schroy et al. indicate that patients have a preference for faecal testing for routine screening due to its convenience and non-invasive nature [52]. Another study by Hol et al. concluded that screening using endoscopy techniques were preferable amongst a screening population due to the certainty of diagnosis and reduction in risk of disease [53]. This indicates that to be successfully implemented in the community, high sensitivity and specificity to accurately and reliably diagnose CRC and inform patient follow-up procedures are important features of any diagnostic test that must be considered.

## Supporting Information

S1 Fig Scatter plots for the seven biomarkers evaluated in the training and test cohorts. (PDF)

S2 Fig Receiver operator characteristic curves for the seven biomarkers evaluated in the training and test cohorts. (PDF)

S1 Table Performance characteristics of the individual biomarkers in the training and test cohorts. (PDF)
