# Supplementary figures and images for: Blood-Based Protein Biomarker Panel for the Detection of Colorectal Cancer
Source: PLoS One. 2015 Mar 20;10(3):e0120425. doi: 10.1371/journal.pone.0120425 (PMC4368610; doi:10.1371/journal.pone.0120425)

**S1 Fig. Scatter plots for the seven biomarkers evaluated in the training and test cohorts.**

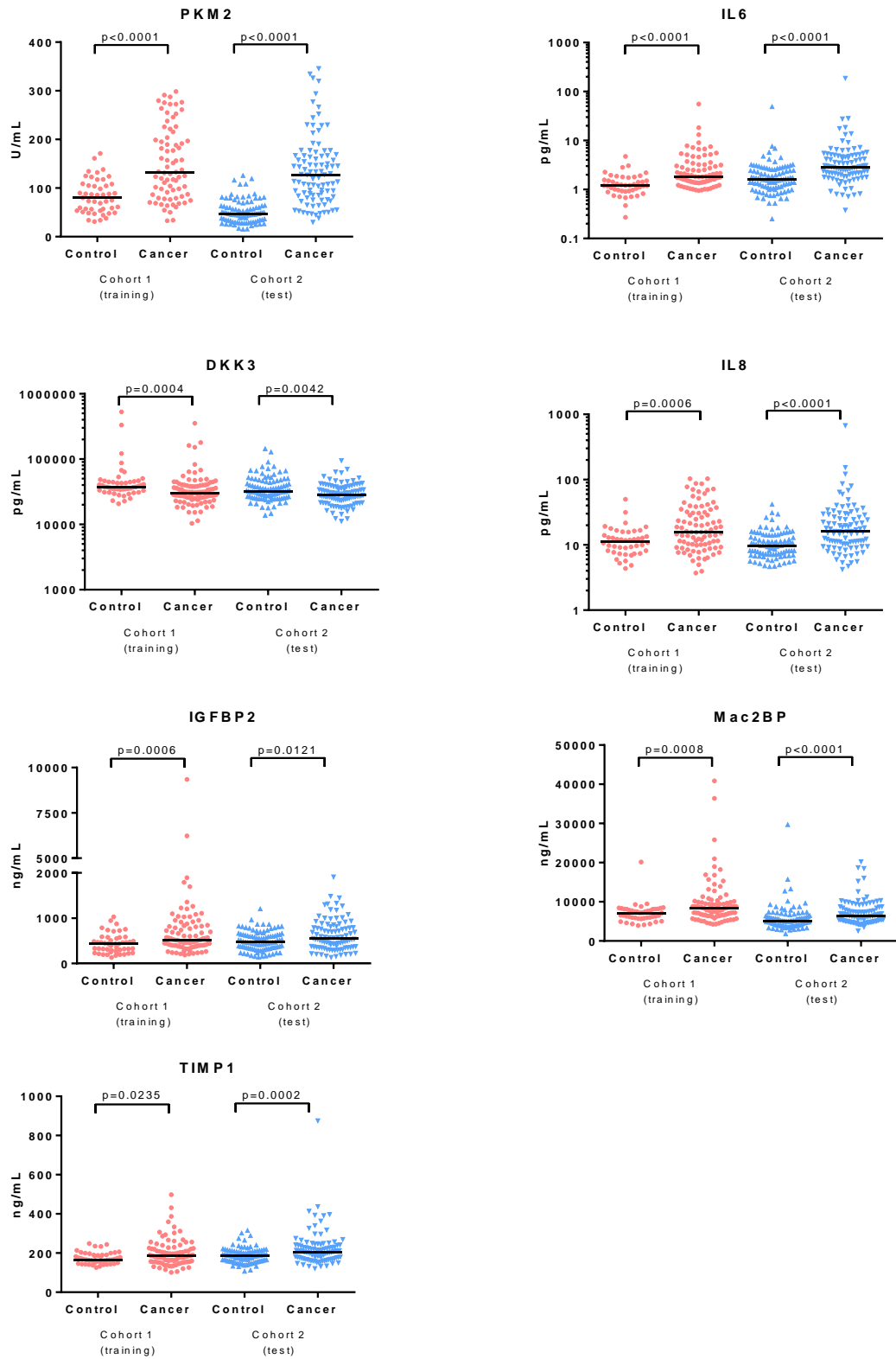

Supplement: S1 Fig — (PDF) [file pone.0120425.s001.pdf]
