# Supplementary material for: Blood-Based Protein Biomarker Panel for the Detection of Colorectal Cancer
Source: PLoS One. 2015 Mar 20;10(3):e0120425. doi: 10.1371/journal.pone.0120425 (PMC4368610; doi:10.1371/journal.pone.0120425)
Supplement: S1 Table — (PDF) [file pone.0120425.s003.pdf]

S1 Table. Performance characteristics of the individual biomarkers in the training and test cohorts.

	DKK3		PKM2		Mac2BP		IGFBP2	
	Training	Test	Training	Test	Training	Test	Training	Test
**Stage I**								
**AUC**	0.63 (0.47-0.76)	0.65 (0.54-0.76)	0.75 (0.60-0.88)	0.88 (0.81-0.94)	0.64 (0.48-0.79)	0.72 (0.61-0.82)	0.64 (0.49-0.78)	0.54 (0.42-0.67)
**P value**	0.0978	0.0142	0.0008	<0.0001	0.0733	0.0005	0.0665	0.5038
**Sens @ 95% Spec**	14	11	48	52	29	15	24	19
**Stage II**								
**AUC**	0.66 (0.52-0.79)	0.57 (0.45-0.68)	0.81 (0.70-0.91)	0.92 (0.85-0.97)	0.66 (0.52-0.79)	0.73 (0.63-0.82)	0.70 (0.58-0.81)	0.59 (0.46-0.71)
**P value**	0.0145	0.2619	<0.0001	<0.0001	0.0212	0.0001	0.0028	0.1364
**Sens @ 95% Spec**	17	10	52	65	38	16	17	26
**Stage III**								
**AUC**	0.74 (0.62-0.85)	0.59 (0.46-0.71)	0.84 (0.75-0.92)	0.92 (0.85-0.97)	0.71 (0.59-0.83)	0.68 (0.57-0.79)	0.61 (0.48-0.73)	0.66 (0.53-0.78)
**P value**	0.0002	0.1645	<0.0001	<0.0001	0.0012	0.0029	0.095	0.0084
**Sens @ 95% Spec**	24	7	61	54	39	7	18	21
**Stage IV**								
**AUC**	0.65 (0.42-0.85)	0.74 (0.58-0.88)	0.91 (0.74-1.00)	0.90 (0.73-1.00)	0.68 (0.46-0.88)	0.65 (0.49-0.80)	0.88 (0.77-0.96)	0.64 (0.44-0.83)
**P value**	0.1525	0.0058	<0.0001	<0.0001	0.1159	0.1194	<0.0001	0.1212
**Sens @ 95% Spec**	20	25	75	80	25	10	40	27
**All stages**								
**AUC**	0.68 (0.59-0.76)	0.62 (0.54-0.69)	0.82 (0.74-0.88)	0.91 (0.86-0.94)	0.68 (0.58-0.76)	0.70 (0.63-0.78)	0.67 (0.58-0.76)	0.60 (0.52-0.68)
**P value**	0.0004	0.0042	<0.0001	<0.0001	0.0008	<0.0001	0.0006	0.0122
**Sens @ 95% Spec**	19	11	56	59	35	12	21	23

	TIMP1		IL6		IL8	
	**Training**	**Test**	**Training**	**Test**	**Training**	**Test**
**Stage I**						
**AUC**	0.56 (0.39-0.73)	0.62 (0.48-0.74)	0.66 (0.49-0.81)	0.77 (0.67-0.86)	0.50 (0.35-0.67)	0.73 (0.62-0.84)
**P value**	0.4224	0.0678	0.048	<0.0001	0.9714	0.0002
**Sens @ 95% Spec**	33	15	31	30	10	15
**Stage II**						
**AUC**	0.69 (0.55-0.81)	0.68 (0.56-0.79)	0.71 (0.58-0.83)	0.74 (0.63-0.85)	0.67 (0.54-0.79)	0.70 (0.57-0.82)
**P value**	0.0053	0.0025	0.0027	<0.0001	0.0136	0.0008
**Sens @ 95% Spec**	17	19	24	35	24	35
**Stage III**						
**AUC**	0.67 (0.54-0.79)	0.66 (0.54-0.77)	0.74 (0.63-0.85)	0.74 (0.63-0.84)	0.77 (0.65-0.88)	0.80 (0.71-0.89)
**P value**	0.0088	0.0081	0.0003	0.0001	<0.0001	<0.0001
**Sens @ 95% Spec**	27	11	30	18	55	36
**Stage IV**						
**AUC**	0.60 (0.40-0.78)	0.65 (0.45-0.84)	0.62 (0.39-0.84)	0.71 (0.51-0.88)	0.82 (0.57-0.99)	0.70 (0.48-0.89)
**P value**	0.353	0.0814	0.2866	0.0306	0.0027	0.0424
**Sens @ 95% Spec**	10	17	12	20	75	40
**All stages**						
**AUC**	0.62 (0.52-0.71)	0.65 (0.58-0.73)	0.70 (0.61-0.79)	0.75 (0.68-0.82)	0.68 (0.59-0.77)	0.74 (0.67-0.81)
**P value**	0.0236	0.0002	0.0002	<0.0001	0.0006	<0.0001
**Sens @ 95% Spec**	20	15	27	27	38	30
